# Plant immunity in plant–aphid interactions

**DOI:** 10.3389/fpls.2014.00663

**Published:** 2014-12-01

**Authors:** Maëlle Jaouannet, Patricia A. Rodriguez, Peter Thorpe, Camille J. G. Lenoir, Ruari MacLeod, Carmen Escudero-Martinez, Jorunn I.B. Bos

**Affiliations:** ^1^Cell and Molecular Sciences, The James Hutton InstituteDundee, UK; ^2^Division of Plant Sciences, University of DundeeDundee, UK

**Keywords:** aphids, plant immunity, effectors, host range, non-host resistance

## Abstract

Aphids are economically important pests that cause extensive feeding damage and transmit viruses. While some species have a broad host range and cause damage to a variety of crops, others are restricted to only closely related plant species. While probing and feeding aphids secrete saliva, containing effectors, into their hosts to manipulate host cell processes and promote infestation. Aphid effector discovery studies pointed out parallels between infection and infestation strategies of plant pathogens and aphids. Interestingly, resistance to some aphid species is known to involve plant resistance proteins with a typical NB-LRR domain structure. Whether these resistance proteins indeed recognize aphid effectors to trigger ETI remains to be elucidated. In addition, it was recently shown that unknown aphid derived elicitors can initiate reactive oxygen species (ROS) production and callose deposition and that these responses were dependent on BAK1 (BRASSINOSTERIOD INSENSITIVE 1-ASSOCIATED RECEPTOR KINASE 1) which is a key component of the plant immune system. In addition, BAK-1 contributes to non-host resistance to aphids pointing to another parallel between plant-pathogen and – aphid interactions. Understanding the role of plant immunity and non-host resistance to aphids is essential to generate durable and sustainable aphid control strategies. Although insect behavior plays a role in host selection and non-host resistance, an important observation is that aphids interact with non-host plants by probing the leaf surface, but are unable to feed or establish colonization. Therefore, we hypothesize that aphids interact with non-host plants at the molecular level, but are potentially not successful in suppressing plant defenses and/or releasing nutrients.

## INTRODUCTION

Plants are facing constant threats from pests and pathogens. Nearly half of the one million known insect species feed on plants (Wu and Baldwin, 2010), including aphids. These insects are phloem-feeders that belong to the family Aphididae and order Hemiptera. Over 4000 aphid species have been described and a number of these are known to damage plant health (Dixon, 1998). Aphids are major economic pests that cause yield losses worldwide, especially in temperate regions (Blackman and Eastop, 2000). Damage to plants as a result of aphid infestation can result in water stress, reduced plant growth, wilting, and importantly, these insects can are vectors of economically important plant viruses.

Most aphid species are highly specialized and can only infest plants in a single taxonomic family or few related plant species (Blackman and Eastop, 2000). However, some aphid species are considered polyphagous and are able to infest plants in many families, including important crops. For example, the aphid species *Acyrthosiphon pisum* (pea aphid) can only colonize plant species in the family *Fabaceae*. In contrast, *Myzus persicae* (green peach aphid) can infest over 400 plant species, mostly dicotyledonous plants, in over 40 families (Blackman and Eastop, 2000), including many important crops. Infestations can develop relatively quickly due to an asexual stage in the aphid life cycle allowing for rapid population growth (Blackman and Eastop, 2000). Most aphid species produce multiple asexual generations through parthenogenesis during spring and summer, when secondary hosts, including many crops, are readily available, and enter a sexual life stage in autumn when the days become shorter and the temperature falls. However, the occurrence of the sexual cycle depends on the presence of the primary host, and in milder climates some species are able to survive through winter without a sexual cycle (Leather, 1992; Pons et al., 1992). Also, evidence suggests that some aphid species or lineages are unable to develop any sexual stages and reproduce exclusively by parthenogenesis (Margaritopoulos et al., 2002; Nibouche et al., 2014).

Current aphid control strategies rely on the frequent use of insecticides in the field. There are an increasing number of restrictions in place on the use of insecticides under European Union (EU) legislation due to their negative impact on the environment. Another major issue is the emergence of new aphid genotypes that have acquired resistance to many of the different types of available insecticides. Different mechanisms can underlie the development of resistance including changes in the cuticle (Ahmad et al., 2006; Zhang et al., 2008), point mutations in insecticide target genes (Li et al., 2007), and increased production of metabolic enzymes that can break down insecticides (Puinean et al., 2010; Jackson et al., 2011). Although insecticide resistance mediated by these mechanisms plays a major role in infestations of field crops, these resistance mechanisms are not the sole determinants of aphid survival in the field and other factors are predicted to be involved (van Toor et al., 2013). With increasing issues related to the use of insecticides, there is an urgent need to explore novel avenues for controlling aphid infestations. To create such avenues there is a need to understand the molecular interplay during plant–aphid interactions in much greater detail.

Traditionally, plant–aphid interactions research has been focused on the plant side, which has provided insights into some of the plant defense mechanisms effective against aphids. These include preformed barriers, constitutive chemical defenses, as well as direct and indirect inducible defenses (Wu and Baldwin, 2010). Recent advances in the study of plant–aphid interactions suggest a complex molecular interplay is taking place. Therefore, a change of perspective is needed to fully understand the molecular basis of susceptibility and resistance. For a plant pathogen or pest to be successful, manipulation of host cell processes to promote virulence is essential, which is achieved by the secretion of molecules termed effectors The identification and characterization of plant pathogen effectors and their host targets to understand their role in promoting virulence has become a hot topic over the past decade (Bos et al., 2010a; McLellan et al., 2013; Gimenez-Ibanez et al., 2014; King et al., 2014). A number of studies have now shown that insects, including aphids, produce and secrete effectors that modulate plant defense responses (Bos et al., 2010b; Atamian et al., 2013; Elzinga and Jander, 2013; Pitino and Hogenhout, 2013). This points to parallels between plant–pathogen and plant–insect interactions and adds a new dimension of complexity to our views on how plants and insects interact at the molecular level.

In this review, we will discuss the latest progress in understanding the molecular basis of plant–aphid interactions. More specifically, we will discuss the latest findings regarding plant immunity and aphid effectors, and highlight the potentially common concepts of plant–pathogen and –aphid interactions. In our discussion we will take a comprehensive view on potential molecular mechanisms involved in both host- and non-host-interactions. In addition, we will discuss the translational opportunities that may arise from our understanding of the plant immunity system and how new approaches could help achieve broad-spectrum and durable aphid resistance in crops.

## HOW DO APHIDS INTERACT WITH HOST AND NON-HOST PLANTS?

The host range of aphid species varies widely, and even among aphid species within the same genera (Blackman and Eastop, 2000). For example, while *M. persicae* has an exceptionally broad host range, its close relative, *M. cerasi,* will only infest a small number of herbaceous plants. The mechanisms underlying such differences in host range remain elusive and most research to date has been focused on understanding the interaction with compatible hosts.

**FIGURE 1:**
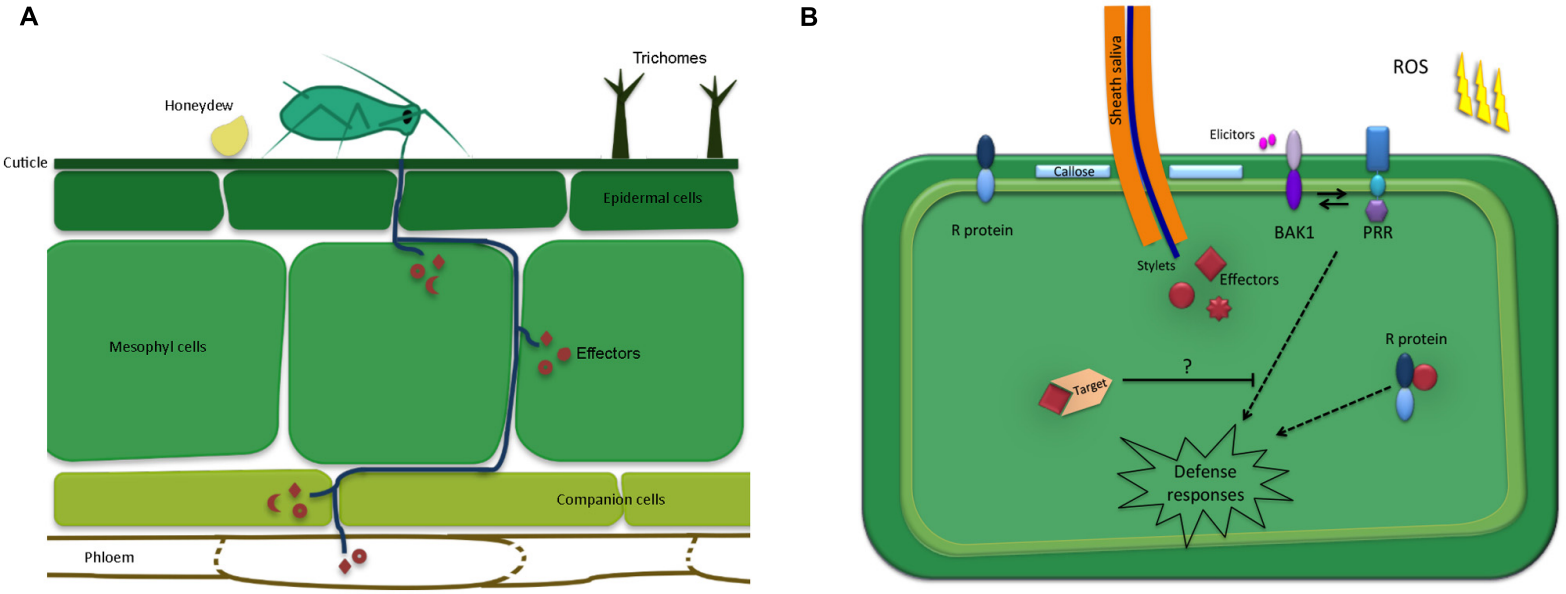
**Schematic overview of the plant-aphid interface. (A)** Aphid mouthparts penetrate the leaf surface upon encountering preformed defenses such as trichomes and waxes. The aphid stylets follow a mainly extracellular pathway while probing and locating the phloem. Most cells along the stylet-pathway are punctured, including the phloem cells. Saliva, containing effectors is secreted into the different cellt ypes as well as the apoplast. In addition, aphids secrete honeydew on the leaf surface, which may also contain molecules that alter plant defense responses.** (B)** Upon probing, aphids secrete effectors inside the host cell cytoplasm, which interact with targets to modulate host cell processes. In resistant plants, these effectors may be recognized by resistance (R) proteins leading to effector-triggered immunity. In addition, the plant may perceive conserved aphid-derived molecules, or elicitors, by means of pattern recognition receptors (PRRs). This perception involves BAK1, which is known to interact with identified PRRs to induce defense responses, including callose formation and the production of reactive oxygen species (ROS) production.

There are several steps involved in the initial contact between winged aphids and plants, prior to any feeding attempts. As detailed by Powell et al. (2006), aphids first need to land on the plant surface, which may be affected by several plant cues, including volatiles. Upon landing these insects may perceive plant cues and structures present on the leaf surface, such as trichomes and waxes, that may affect their behavior. In compatible plant–aphid interactions aphids feed from sieve tube elements in the phloem using specialized mouthparts, called stylets, to obtain nutrients essential for survival and reproduction. However, aphids will attempt to penetrate the leaf surface using their stylets regardless of the plant species. It is thought that this probing behavior also generally takes place in aphid-non-host interactions and is responsible for the high transmission rates of viruses by aphids on non-host plant species (Powell et al., 2006). Importantly, these observations indicate that there is an opportunity for molecular interactions to take place during both aphid-host and – non-host interactions. However, during non-host interactions aphids are either unable to reach the phloem or unable to successfully feed from the phloem. Probing and feeding behavior during compatible plant–aphid interactions has been well documented and is thought to involve cues such as pH, sucrose and amino acid content (Tjallingii, 2006; Hewer et al., 2011; Will et al., 2013). The aphid stylets follow a mainly extracellular pathway through the apoplast, but briefly puncture most cells along the pathways in the epidermis, mesophyll and vascular tissue. Saliva is secreted as soon as these insects make contact with the plant tissue and continues throughout the probing and feeding process (Moreno et al., 2011). During this constant interaction with their hosts, plant defenses are likely to be triggered. Such defenses are thought to be suppressed in host plants by the delivery of aphid effectors secreted within the saliva (**Figure 1A**).

Although recent work has started to unravel some potential mechanisms involved in (non)host resistance to aphids, this area of research is currently understudied with many questions remaining unanswered. For example, how do crops actually respond to aphids in incompatible interactions? What type of plant defenses are activated, and how are they activated? And are similar defenses involved across different crops? On the aphid-side of the interaction, what genes contribute to aphid host range, and how? Addressing these questions is essential for crop improvement and long-term protection against aphids, and potentially other insect pests.

## DEFENSE SIGNALING DURING PLANT–APHID INTERACTIONS

Plants use preformed barriers such as trichomes, hairs, and waxes as a layer of defense against herbivore attacks (Hanley et al., 2007; War et al., 2012; Fürstenberg-Hägg et al., 2013; **Figure 1**). In some pathogen/pest interactions these barriers may be sufficient to confer resistance. However, it is likely that in many interactions complex series of molecular events are involved. Most studies of plant defense signaling in plant–aphid interactions have focused on the model system *Arabidopsis thaliana-Myzus persicae*. This revealed the contribution of several signaling pathways involved in activating defenses. For example, *M. persicae* feeding was found to induce the expression of SA-signaling pathway marker genes, such as PR-1 (Moran and Thompson, 2001; De Vos et al., 2005). Recent work from Kerchev et al. (2013), aimed to compare local versus systemic gene expression patterns upon *M. persicae* feeding on *Arabidopsis*. This showed that in both local and distal leaves SA-, ET-, and ABA (abscisic acid)-signaling genes were induced, while JA-responsive genes were repressed. It has been speculated that the activation of SA-signaling pathways by aphids counters the activation of JA-dependent defense responses that are effective against aphids (De Vos et al., 2007). However, high levels of SA-signaling do not necessarily correspond with increased susceptibility to aphids. For example, *Arabidopsis* mutants with low ascorbate levels show increased SA-signaling, but show a decrease in susceptibility to *M. persicae* (Kerchev et al., 2013). Thus, the activation of SA-signaling does not necessarily suppress JA-dependent aphid defenses to promote virulence.

*Arabidopsis* mutant analyses indicated a role for several defense signaling pathways in the interaction with *M. persicae*. While Mewis et al. (2005) showed that both *npr1* mutant and *NahG* transgenic *Arabidopsis* lines were less susceptible to *M. persicae*, this difference was not found by Pegadaraju et al. (2005) who reported no difference of these lines to the control. Whether these contrasting findings reflect minor effects or differences in experimental conditions remains to be resolved. Nonetheless, more detailed studies on SA signaling pathways during aphid infestation of a range of host plants should help illuminate the role of SA-mediated defense in plants. Evidence for a role of JA in the activation of defenses against *M. persicae* was provided by Ellis et al. (2002) who showed that *Arabidopsis* mutants that show a constitutive activation of JA-signaling were more resistant to aphids than wild-type plants. Interestingly, and despite changes in marker gene expression, no increase in detectable levels of SA, JA, or ET was detected in *Arabidopsis* plants infested with *M. persicae* (De Vos et al., 2005), suggesting that regulation of marker gene expression and defense signaling may be more complex and does not solely rely on the production of these hormones. Defense against aphids also relies on components that act independent from hormone signaling pathways. For example, *PAD4*, which encodes a lipase-like protein with a role in plant immunity, contributes to defense responses against *M. persicae*, with aphids reproducing more on *pad4* mutants, but less on *PAD4* overexpressing lines (Pegadaraju et al., 2007). Interestingly, the contribution of *PAD4* to this defense was found to be phloem-based and independent of *EDS1* and SA-signaling (Pegadaraju et al., 2005, 2007). Another component found to contribute to defense responses against *M. persicae* is *PAD3*, (Kettles et al., 2013), which encodes a cytochrome P450 that is involved in the biosynthesis of the phytoalexin camalexin. Interestingly, the plant miRNA pathway was shown to regulate *PAD3* expression, and consequently, the production of camalexin. More specifically, *PAD3* is more highly expressed in the *dcl1* mutant, which is affected in the miRNA processing pathway. This induction corresponds with an increase in camalexin levels and a reduction in aphid performance. Other secondary metabolites with a role in defense against *M. persicae* include glucosinolates. Aphid feeding triggers the accumulation of indole glucosinolates (Kim and Jander, 2007). Moreover, mutants with increased levels of indol-3-ylmethylglucosinolate were less susceptible to aphids, while mutants with decreased levels of indole glucosinolates collapsed more rapidly upon aphid attack (Kim et al., 2008).

## PLANT IMMUNITY TO APHIDS

Despite the activation of host defenses, *M. persicae* is able to overcome these to successfully feed from and reproduce on an impressively wide range of plant species. While research on plant–aphid interactions has long been focused on the plant-side, with resistance genes, hormones and secondary metabolites being discovered, there has been a recent shift to the aphid-side of the interaction. It is evident that aphids are able to manipulate host responses, as reflected by their ability to affect host morphology (van Emden and Harrington, 2007), impact host nutrient allocation (Girousse et al., 2005), and suppress plant defense responses (Will et al., 2007). This implies there is an active interplay between plants and aphids at the molecular level. The emerging aphid effector paradigm suggests that aphids, like other plant-associated organisms secrete effectors inside their host to manipulate host cell processes through the interaction with host molecules (Elzinga and Jander, 2013; Rodriguez and Bos, 2013). In this case, the plant–parasite interaction becomes a complicated molecular-warfare battle, where an evolutionary “arms race” takes place. A zig-zag model has been proposed to model the interaction that takes place between plants and parasites (Jones and Dangl, 2006). However, to what extend the model would apply to aphids, and potentially other insects, is yet unknown. According to the model, aphids present conserved molecules similar to PAMPs (Pathogen Associated Molecular Patterns) that trigger PAMP-triggered immunity (PTI; Hogenhout and Bos, 2011; Rodriguez and Bos, 2013). Aphids then deliver effectors inside their host to suppress this as well as other defenses to promote effector-triggered susceptibility (ETS). Some plant species/genotypes may carry receptors, or R (Resistance) proteins that can recognize these effectors leading effector-triggered immunity (ETI). In reality the plant defense system is expected to be more complex than the zig-zag model implies and there may be overlap between the molecular requirements for PTI and ETI (Thomma et al., 2011). A state based model has also been proposed where the plant monitors the “state” of the cell for a change that differs from normal. Any such change will result in a response depending on the state change (Pritchard and Birch, 2011). However, for the purpose of this review the plant defense system will be discussed in the context of the zig-zag model.

As mentioned above, aphids may encounter an array of plant defense responses upon dealing with any preformed barriers/defenses, including PTI. As reviewed by Dodds and Rathjen (2010), PTI can result in (1) chemical defense, e.g., production of reactive oxygen species (ROS), (2) structural defense, e.g., cross-linking of the cell wall and deposition of callose, (3) signaling, e.g., activation of mitogen-activated protein kinases (MAPK), reprogramming of gene expression, hormone signaling and systemic signaling to activate defense responses in neighboring cells. Examples of PAMPs include peptidoglycan, and lipopolysaccharides, oomycete glucans, bacterial flagellin, and chitin, a component of the fungal cell wall (Felix et al., 1993, 1999; Dow et al., 2000; Vleeshouwers et al., 2006). Elicitors of plant immunity have been identified in several herbivorous insects, some of which function indirectly causing plant damage and generation of DAMPS (Damage Associated Molecular Patterns as reviewed by Hogenhout and Bos (2011).

To date no elicitor has been identified as an aphid HAMP (Herbivore Associated Molecular Pattern). However, elicitor activity has been detected in both aphid saliva and whole aphid extracts. For example, an unknown elicitor present in the 3–10 kDa fraction of saliva from the aphid species *M. persicae* induces defense responses in *A. thaliana* effective against aphids (De Vos and Jander, 2009). The activation of defenses by aphid saliva was independent of JA, SA, or ET signaling molecules, and only few JA or SA signaling pathway-related genes were induced upon saliva treatment, suggesting alternative defense mechanisms may be involved.

Additional evidence for the activation of immunity by aphids is provided by a recent study where infiltration of whole aphid extract from *M. persicae* activated PTI-like responses in *Arabidopsis* (Prince et al., 2014). While both the 3–10 and >10 kDa fractions induced resistance to aphids, only the 3–10 kDa fraction triggered the ROS burst, suggesting multiple elicitors were present in the whole aphid extracts. The activation of PTI-like responses was dependent on BAK1 (**Figure 1**), as *bak1* mutants showed an increased survival rate of the pea aphid on *Arabidopsis* (non-host interaction), and an increased susceptibility to *M. persicae* (host interaction). In addition, induced resistance required PAD3, indicating at least two separate signaling pathways are involved. It is possible that the elicitors responsible for the observed activation of PTI-like responses are derived from the aphids themselves or any of the organisms that are associated with the aphids, such as bacteria. Indeed, work by Chaudhary et al. (2014) provides evidence for the latter. Using mass spectrometry on saliva from the aphid species *Macrosiphum euphorbiae* they showed the presence of the chaperonin GroEL from the aphid primary endosymbiont *Buchnera aphidicola.* These endosymbionts are located in specialized compartments called bacteriocytes in the hemocoel of the insects and contribute to the production of amino acids for their host. Although these bacteria strictly reside within aphids, it is interesting they are able to secrete proteins that are delivered with aphid saliva inside plants and activate defenses. Infiltration of purified GroEL protein resulted in the induction of PTI-like responses in *Arabidopsis.* More specially, GroEL induced callose deposition and ROS in a BAK1 dependent manner. Also, overexpression of GroEL in tomato and *Arabidopsis* reduced aphid fecundity. Overall, these recent findings suggest that the co-receptor BAK1, an important player in PTI mediated by plant pathogenic bacteria, is involved in perception of aphids and/or aphid-associated microbes. Interestingly, not only the saliva, but also the honeydew of aphids can alter plant defenses (Schwartzberg and Tumlinson, 2014) and has been found to contain bacterial proteins (Sabri et al., 2013). These include EF-Tu, chaperone proteins, and flagellin, which are derived from the aphid microbiota (Sabri et al., 2013). The microbiota of aphids is thought to not only consist of primary and secondary endosymbionts, but also plant pathogenic bacteria such as *Pseudomonas syringae* (Stavrinides et al., 2009), as well as a range of other bacteria, including *Staphylococcus* species, *Serratia marcescens* and *Erwinia* (Sabri et al., 2013). Whether these aphid-associated microbes are all able to trigger plant defenses and the impact of this on plant–aphid interactions remains to be investigated. In another herbivore species, the Colorado potato beetle, secretion of symbiotic bacteria is used as a mechanism to divert and suppress plant defense responses directed against the insect (Chung et al., 2013). Therefore, depending on the insect species, activation of plant immunity by bacterial proteins may contribute to or prevent activation of defense responses.

Chitin is a known fungal PAMP, but also is an important structural component of the aphid stylets and exoskeleton (Uzest et al., 2010). The LysM receptor CERK1 (Chitin Elicitor Receptor Kinase1) is required for the perception of fungal chitin (Miya et al., 2007) and functions independently of BAK1 (Zipfel, 2009). However, *Arabidopsis fls2 erf cerk1* triple mutants were able to produce a ROS response upon treatment with whole aphid extracts indicating that the elicitor responsible for the observed activation of PTI–like responses was not detected by these PRRs (Prince et al., 2014). During probing and feeding, aphid mouthparts/ stylets are put directly in contact with the plant cell surfaces. It is therefore possible that plants have evolved ways of detecting core structural components, including chitin as non-self molecules. It has been proposed that a gel-like form of saliva is secreted to coat the stylets during probing and feeding to prevent extracellular perception and/or signaling associated with the detection of the stylets (Will and van Bel, 2008). Whether or not chitin is involved in aphid perception, potentially by a currently unidentified PRR, remains to be investigated.

Another form of plant immunity, ETI, is activated upon recognition of parasite effectors by resistance genes. Gene-for-gene type interactions have been found in several aphid-host systems. For example, extensive variation in the ability of *A. pisum* biotypes to infest *Medicago* genotypes has been observed (Kanvil et al., 2014). Also, biotypes of *Diuraphis noxia* have emerged that come overcome resistances in wheat in what appears to be a gene-for-gene specific manner (Haley et al., 2004; Burd et al., 2006). Other systems where aphid biotypes can overcome important crop resistances that have been used in breeding include *Nasonovia ribisnigri*-lettuce (ten Broeke et al., 2013) and *Aphis glycines*-soybean (Kim et al., 2008). Similar to ETI in plant pathogen interactions, it is likely aphid effectors are recognized by R proteins leading to plant resistance, and that mutations and or changes in expression of such proteins occur in biotypes able to overcome host resistance (**Figure 1B**).

Aphid resistances have been identified in many cultivated crops, and for some of these the gene responsible for conferring resistance has been identified (Dogimont et al., 2010). The tomato *R*-gene *Mi-1.2* belongs to the class of NB-LRR proteins and confers resistance to specific *Macrosiphum euphorbiae* (potato aphid) genotypes (Rossi et al., 1998). Interestingly, the same R protein also confers resistance to root-knot nematodes (RKN; Rossi et al., 1998), whitefly (Nombela et al., 2003) and psyllids in tomato (Casteel et al., 2006). On *Mi-1* resistant plant, aphids showed increased probing attempts, suggesting that sieve element ingestion was potentially limited (Kaloshian et al., 2000). Transgenic eggplant expressing *Mi-1* was resistant to RKN but not to *M. euphorbiae* indicating different requirements for *Mi-1* mediated resistance to nematodes and aphids (Goggin, 2007). Other *R* genes belonging to the NB-LRR family are Vat from melon, which confers resistance, involving a hypersensitive response, to *Aphis gossypii* (Villada et al., 2009) and Ra, which confers resistance to the lettuce-root aphid, *Pemphigus bursarius L* (Wroblewski et al., 2007). Although, *R* genes have been identified that confer resistance to some aphid species, the effectors responsible for recognition remain elusive.

With evidence for both a role of PTI and ETI in plant–aphid interactions it is essential to further investigate the genes involved in activation of signaling pathways and immunity. In addition, an open mind is needed with regards to the potential defenses that may be relevant to host and non-host resistance in different plant–aphid systems. It is possible that other mechanisms, in addition to PTI and ETI, are key in activating defenses against aphids.

We have currently limited insight into the biological processes taking place in not only host, but also non-host interactions with aphids. Questions driving future research include: what range of defense responses do aphids need to suppress to be able to establish infestations? What cell biological processes are affected by aphids during interactions to promote susceptibility, and how does this compare to what we know in interactions with other plant-associated organisms? What are the early and late plant responses that either determine susceptibility or resistance? And how do these compare among different plant species, including crops and among interactions with different aphid species? By addressing these questions we will acquire a better fundamental understanding of how aphids and plants interact at the molecular levels, which in turn will allow development of novel aphid effector activity assays for effector identification and characterization. Moreover, such insights are needed to understand the full range of plant defenses that act against aphids not only in susceptible but also, importantly, in resistant plants.

## APHID EFFECTOR PROTEINS

Aphid effectors are most likely expressed in salivary glands and secreted into saliva, which is delivered inside the host during feeding and probing (Hogenhout and Bos, 2011; Elzinga and Jander, 2013). Recent availability of aphid genomic resources and proteomics tools has allowed the identification of considerable sets of predicted candidate effectors from several aphid species as reviewed by Elzinga and Jander (2013) and Rodriguez and Bos (2013). Among the identified (predicted) secreted salivary proteins, or candidate effectors, to date some have predicted activities. These include cell wall-degrading enzymes (pectinases, glucanases, amylases) and detoxifying enzymes (oxidoreductases, phenol oxidases, peroxidases; Harmel et al., 2008; Carolan et al., 2009, 2011; Cooper et al., 2010, 2011; Cui et al., 2012; Nicholson et al., 2012; Rao et al., 2013). However, a large number of candidate effectors have no similarity to proteins of predicted function, some of which are specific to aphids. Only a limited number of these effector proteins have been characterized to date and have been implicated in promoting or decreasing virulence and activating or suppressing defenses (**Table 1**).

**Table 1:** Summary of currently characterized aphid effector proteins.

Effector	Aphid species	Role	Molecular activity	Reference
Mp55	*Myzus persicae*	Suppression of plant defenses	Lower accumulation of 4-methozyindol-3-ylmethylglucosinolate, callose and H_2_O_2_	Elzinga and Jander (2013)
COO2	*Acyrthosiphon pisum*; *Myzus persicae*	Essential for aphid feeding	So far unknown	Mutti et al. (2006, 2008), Bos et al. (2010b), Pitino et al. (2011)
Mp1/PIntO1	*Myzus persicae*	Enhanced aphid fecundity	So far unknown	Bos et al. (2010b), Pitino and Hogenhout (2013)
PIntO2	*Myzus persicae*	Enhanced aphid fecundity	So far unknown	Pitino and Hogenhout (2013)
Mp10	*Myzus persicae*	Reduced aphid fecundity	Altering JA- and SA-defense related signaling in plants	Bos et al. (2010b), Rodriguez et al. (2014)
Mp42	*Myzus persicae*	Reduced aphid fecundity	Perturbation of nuclear envelope and membranes; aggregate formation in ER	Bos et al. (2010b), Rodriguez et al. (2014)
Me23	*Macrosiphon eurphorbiae*	Enhanced aphid fecundity	So far unknown	Atamian et al. (2013)
Me10	*Macrosiphon eurphorbiae*	Enhanced aphid fecundity	So far unknown	Atamian et al. (2013)

Functional characterization studies have identified several aphid candidate effectors that, upon over-expression, negatively impact aphid virulence. This could indicate such proteins activate defenses against aphids, or observations can results from the high expression levels that are not representative of the natural system (for example, excessive targeting of a host target may take place). Both Mp10 and Mp42 were identified from a functional genomics screen, and reduced aphid virulence upon overexpression in *N. benthamiana* (Bos et al., 2010b). Only Mp10 induced chlorosis and local cell death, and suppressed the ROS burst triggered by flg22 but not chitin. Further characterization of Mp10 and Mp42 showed that these proteins exhibit different intracellular activities, probably toward distinct cellular targets (Rodriguez et al., 2014). For instance, Mp10 and Mp42 have different subcellular localization profiles when transiently overexpressed in *N. benthamiana*, and only Mp10 activates hormone-related defense signaling and reduces host susceptibility to the oomycete *P. capsici* (Rodriguez et al., 2014). However, Mp10 also appeared to affect *Agrobacterium*-mediated overexpression of proteins, making it difficult to investigate Mp10 function using overexpression assays. It is possible that Mp10 has dual activities in both activating and suppressing different types of host defense responses, or that the observed activation and suppression of host defences are somehow linked.

A number of aphid effectors identified to date are able to promote aphid virulence upon transient and/or transgenic over-expression. The effector C002, which was first identified in *A. pisum*, is an abundant salivary protein that is essential for aphid feeding (Mutti et al., 2008). When C002 transcription is reduced by RNA interference (RNAi) in the pea aphid, aphid lethality increases as aphids have difficulties reaching the sieve tube elements (Mutti et al., 2006, 2008). Further characterization of the C002 ortholog from *M. persicae* (MpC002) showed this protein promotes aphid virulence upon overexpression in *N. benthamiana* leaf discs (Bos et al., 2010b). Similar observations were made when MpC002 was overexpressed in transgenic *Arabidopsis* lines (Pitino and Hogenhout, 2013). Interestingly, when *M. persicae* feeds from plants overexpressing the pea aphid form of C002, their progeny is not affected (Pitino and Hogenhout, 2013). Besides variation at the amino acid level between MpC002 and ApC002, there is variation in the presence of an N-terminal repeat region. Removing this region from *Mp*C002 abolished its ability to promote *M. persicae* virulence (Pitino and Hogenhout, 2013). It is possible the MpC002 repeat region is important for protein stability and/or function.

Other *M. persicae* effectors able to promote aphid virulence include Mp1/PIntO1 and PIntO2 (Bos et al., 2010b; Pitino and Hogenhout, 2013). Mp1/PIntO1 is also an abundant protein in *A. pisum* saliva (Harmel et al., 2008; Carolan et al., 2009) and recent data showed that *M. persicae* performed better on *Arabidopsis* plants overexpressing *Mp1/PIntO1* and *MpPInt02*, but not on plants overexpressing a similar sequences from *A. pisum*, *ApPIntO1,* and *ApPIntO2* (Pitino and Hogenhout, 2013). This suggests aphid effectors may function in a host species-specific manner. Recently, Elzinga et al. (2014) have functionally characterized a *M. persicae* effector called Mp55. They showed that Mp55 was able to promote aphid virulence upon overexpression in transgenic *Arabidopsis* lines. These transgenic plants showed a decreased accumulation of 4-methoxyindol-3-ylmethylglucosinolate, callose, and H_2_O_2_ in response to aphid feeding as compared to wild type plants. Silencing of *Mp55* in aphids using RNAi led to a reduction of virulence on *N. tabacum*, *A. thaliana,* and *N. benthamiana*. Although the molecular function of Mp55 is not known yet, evidence suggests a role of Mp55 in suppression of plant defenses during the interaction of *M. persicae* with several host species (Elzinga et al., 2014). In addition, several aphid effectors from the aphid species *M. euphorbiae* have been shown to impact plant–aphid interactions (Atamian et al., 2013). Both aphid candidate effectors Me10 and Me23 enhanced aphid virulence when transiently overexpressed in *N. benthamiana*. Me10, but not Me23, also enhanced virulence upon overexpression in tomato using a *Pseudomonas syringae* type three-secretion system (TTSS).

With aphid candidate effector lists being reported for an increasing number of aphid species, including pests of dicot and monocot plants, tool development to assay these proteins for activities is a priority. As mentioned above, transgenic *Arabidopsis* lines as well as transient over-expression systems in both *N. benthamiana* and tomato have been successfully used to identify effector activities. In addition, more recently developed transient expression systems, such as the *P. fluorescens* Effector-to-Host Analyzer (EtHAn) system (Thomas et al., 2009; Upadhyaya et al., 2014), where non-pathogenic bacteria are engineered to express the *P. syringae* TTSS, may be used to characterize aphid candidate effectors in non-model crops such as wheat, barley, or even legumes.

High-throughput screening for relevant effector activities is an effective approach to prioritize effectors for functional characterization to understand their role in promoting susceptibility. The next step is to move forward toward host target identification, which can be achieved using several protein-protein interaction approaches, such as Yeast-two-Hybrid screening and immunoprecipitation of protein complexes followed by mass spectrometry. Identification of host targets and characterizing their role in plant–aphid interactions, and potentially in aphid host range, will be an important step forward toward developing novel control strategies against aphids.

Another interesting aspect is variation of aphid effector repertoires. Interestingly, work by (Cooper et al., 2010) suggested that aphid species causing similar damage to shared host plant species may share salivary protein profiles. This raises the question whether differences in aphid effector repertoires reflect the variation in aphid host range. It is possible that, like other plant parasites, aphids require a core set of proteins for the infestation process in general, but unique sets of proteins to deal with host species-specific defenses and/or other cellular processes to allow infestation. It will be interesting to compare repertoires among the different species, and within species among genotypes, in more detail to determine whether there are core and specific sets of effector proteins that may contribute to virulence and potentially to interactions with specific host plant species.

In addition to effectors, there are likely to be other classes of aphid proteins that are involved in plant infestation and potentially impact aphid host range. These could include digestive enzymes that help determine whether aphids can successfully feed from a particular host plant, or proteins involved in olfaction. Moreover, primary and secondary symbionts, viruses, and even plant pathogenic bacteria associated with aphids could add another level of complexity to plant–aphid interactions. There is the possibility that such organisms are involved in suppression and/or activation of a range of host responses during plant aphid interactions and affect susceptibility or possibly host range.

## HOW CAN CURRENT RESEARCH CONTRIBUTE TO DURABLE APHID RESISTANCE IN CROPS?

As mentioned above, current aphid control heavily relies on the use of insecticides. Traditionally the control of pest insects has focused on mortality as a measure of efficiency. However, if we consider aphid fitness, there are many aspects by which we can measure the efficiency of control methods. Growth and development, reproduction, nutrient acquisition and survival are all vital factors determining the success of an aphid population. Negatively affecting one or many of these factors will likely reduce the overall fitness of an individual or population. Effective control does not necessarily have to rely on aphid mortality, especially if being used in the context of integrated pest management (IPM). In general, IPM relies on the use of multiple control methods simultaneously (e.g., crop rotation, biological control, breeding, transgenic plants). By applying multiple measures to control a pest, it is less likely that new strains, biotypes or isolates may occur that overcome crop resistance, or that develop pesticide resistance. Research aiming to decipher the mechanisms by which pests interact with their hosts plays a crucial role in IPM strategies.

One alternative to the application of insecticides to control aphid population growth is the deployment of biological control agents. Aphids have a range of natural predators that can help bring down population size or prevent populations from expanding. Predators used for biocontrol of aphids include predators like lady beetles (Coleoptera: Coccinellidae), parasitic wasps (Hymenoptera: Braconidae), and entomopathogenic fungi.

In several plant–aphid species interactions the use of (in many cases dominant) *R* genes has been successful in preventing infestations in the field. Resistances have been effectively used for years in many crops including soybean, lettuce, melon, tomato, wheat, barley, maize, legumes, as well as fruit trees (Dogimont et al., 2010). However, over the past decades it has become apparent that aphids are able to overcome such resistance with the emergence of new biotypes. In some crops, where multiple resistances are available, stacking of R genes may be more effective in controlling aphids than deploying single genes (Wiarda et al., 2012). However, a biotype of *A. glycines* able to overcome a pyramid of resistances has recently appeared (Alt and Ryan-Mahmutagic, 2013), indicating aphids may be highly adaptable to such an approach. The molecular mechanisms that underlie the activation of host resistances and emergence of new biotypes remain elusive. By identifying the aphid genes that are responsible for *R* gene activation, and understanding the signaling pathways involved in downstream defenses, we may be able to develop more informed resistance breeding strategies. However, one limitation here is that aphid resistance may not be available in certain crops, especially in the case of broad host range species. Potentially, sources of resistance in such cases may be sought in wild species or alternatively in non-host plants.

The increasing availability of aphid genomics resources generates the opportunity to look for (aphid-specific) genes that may be suitable targets for the development of novel chemical control strategies that act specifically on the target organism. Alternatively, such targets may be used in control strategies based on RNAi. RNAi-mediated gene silencing was achieved in the laboratory by both injection of dsRNA directly into the aphid hemolymph and by allowing insects to feed on an artificial diet containing dsRNA (Mutti et al., 2006; Jaubert-Possamai et al., 2007). Neither method is viable for administering dsRNA to an aphid population in the field. Therefore we must consider alternatives, such as delivery of dsRNA to aphids via the host plant. Pitino et al. (2011) demonstrated that plants expressing dsRNA corresponding to select aphid genes with a predicted role in virulence resulted in silencing of those genes with negative effects on aphid fitness. Thus, it may be possible to generate (more) resistant cultivars for agricultural use by generating transgenic lines expressing specific dsRNAs. A major benefit when considering the use of dsRNA is that they can be designed to act highly specific to the target insect. This means a reduction in the mortality rates of non-target organisms and, most importantly, beneficial insects such as bees or natural enemies of pests.

A highly underexplored area where potential novel sources of aphid resistance may be identified is non-host resistance. Evidence is only now emerging of plant genes that contribute to non-host resistance, and as described above such genes may be involved in activation of PTI or unknown defenses against aphids. With extensive host range variation among different aphid species, it will be interesting to investigate whether and how plants limit aphid host range and whether specific aphid genes, such as effectors, contribute to host range in the case of aphids with broad host ranges. Such insights will be particularly important to understand some of the most economically important aphid species, which have exceptionally broad host ranges, such as *M. persicae*.

With aphids causing significant damage to economically important crops and issues with insecticide resistance and restrictions on their use, there is a need for the development of novel and sustainable aphid control strategies. However, to be able to develop such strategies there is a pressing need to gain a better understanding of the molecular basis of plant–aphid interactions and thus a need for fundamental research on the molecular mechanism involved.

## Conflict of Interest Statement

The authors declare that the research was conducted in the absence of any commercial or financial relationships that could be construed as a potential conflict of interest.
